# Early diagnosis and standardized treatment are critical to improve the prognosis of patients with Takayasu’s arteritis

**DOI:** 10.1515/rir-2024-0001

**Published:** 2024-03-31

**Authors:** Xinping Tian, Xiaofeng Zeng

**Affiliations:** Department of Rheumatology and Clinical Immunology, Peking Union Medical College Hospital, Peking Union Medical College, Chinese Academy of Medical Sciences, National Clinical Research Center for Dermatologic and Immunologic Disease, Ministry of Science& Technology, State Key Laboratory of Complex Severe and Rare Diseases, Key Laboratory of Rheumatology and Clinical Immunology, Ministry of Education, Beijing 100730, China

Takayasu’s arteritis (TAK) is the most common large vessel vasculitis (LVV) in adults although it is regarded as a rare disease from the incidence and prevalence point of view. It mainly affects the aorta and its primary branches resulting in wall thickening, stenosis or occlusion of the affected arteries and aneurysms in a small proportion of patients. The incidence of TAK in Asia young women is much higher than that in western countries. Although there is no epidemiological data in China in recent 20 years, it is estimated that there are at least 50, 000 patients with TAK in China. ^[1]^ Clinical studies have shown that the incidence of comorbidities in TAK patients is significantly increased. According to the data from the Chinese Registry for Systemic Vasculitis (CRSV) cohort,^[2]^ the prevalence is 3.6 % for coronary heart disease, 5.4 % for stroke and 1.3 % for malignancy respectively in a patient population with an average of 29.6 years old at disease onset. TAK has become one of the important causes of premature death in China. In addition, TAK has a significant adverse impact on the quality of life of patients. 74 % of patients reported that their daily life was affected.^[3]^ The 10-year mortality rate of TAK patients is 5 %, but the 10-year mortality rate of patients with severe diseases can be as high as 27%.^[4]^ Therefore, early diagnosis, standardized treatment and manage complications are important to alter the nature course of the disease, improve the overall prognosis and quality of life of patients with TAK. However, the management of patients with TAK in real-life is generally unsatisfactory. Many challenges need to be dealt with when managing TAK in countries and areas where TAK is prevalent.

The clinical manifestations of TAK are heterogenous and very non-specific at early phase, so many patients are mis-diagnosed. Most patients were diagnosed after remarkable organ damage happened. Internationally, the average delay in diagnosis of TAK is 1.4 years,^[5]^ while the data from CRSV cohort showed that the average delay time from the onset of symptoms to definitive diagnosis in TAK patients in China is 2.15 years. The disease is progressive if not intervened. Like the majority rheumatic diseases, early detection and early treatment of TAK can slow down or halt the disease progression, avoid vital organ ischemia caused by severe vascular stenosis, and thus substantially improve the prognosis. Therefore, early diagnosis should be emphasized. Improving early diagnosis rate and shorten the delayed diagnosis time is the first step to reach the final goal of improving the prognosis. Physicians should be alert to TAK when young female presents with unexplained fever accompanied by neck pain. Careful physical examination and vascular imaging examinations should be performed to identify patients with early disease.

TAK is a chronic systemic vasculitis that varies in disease extent and severity. The locations and severity of the involved arteries are closely related to the survival and quality of life. More importantly, the treatment strategy is based on the activity and severity of the disease. Therefore, comprehensive and timely evaluation of disease activity, disease severity and organ damage are essential to make treatment decisions. However, since the acute phase reactants cannot reliably reflect disease activity, especially in patients with minor relapse, careful physical examination with appropriate imaging examinations should be strongly encouraged. But the selection of the most appropriate examination modalities and the interpretation of the examination results are very challenging because some imaging modalities are operator-dependent and are affected by medications used to treat TAK. These may result in incorrect assessment of the disease activity and severity, which lead to undertreatment and overtreatment as a consequence. Therefore, comprehensive evaluation of the disease should be emphasized.

Large arteries involved in TAK are generally accessible by imaging modalities. The arterial wall thickness and lumen diameters could be directly measured, so imaging examinations are important tools for disease severity and activity evaluation. In recent years, traditional angiography has been replaced by computed tomography angiography (CTA). CTA has become the important evidence for the diagnosis of TAK because it can not only demonstrate the extent of the disease, but also the severity of the lesions. Contrast-enhanced ultrasound has emerged as a new technology that can be semi-quantitatively reflect the degree of vascular inflammation by scoring the intensity of contrast intake in the wall of the inflamed arteries. Superb microvascular imaging (SMI), which can directly reflect the pathological basis of TAK, ie, inflammation of the vasa vasorum of the walls of large arteries, has been used to evaluate disease activity recently. Artery magnetic resonance imaging (MRI) can not only show the extent and severity of the affected arteries, but also can directly detect artery wall edema, so it has been regarded as an reliable modality to evaluate disease activity and treatment efficacy. Positron emission tomography (PET) /computed tomography (CT) has been thought to be a surrogate marker of increased activity of inflammatory cells in the artery wall. It’s role in evaluating disease activity of TAK has been established. A quantitative score system has also been developed and has been shown to have good association with disease activity. However, each imaging modality has its own advantages and limitations. Some imaging presentations can be affected by the mediations used in treating TAK. For example, the ^18^F-FDG intake will greatly diminished when being treated with high dose glucocorticoid for 2 weeks. Furthermore, the findings of vascular changes by ultrasound are largely operator dependent. Therefore, rheumatologists must understand the advantages and limitations of each imaging modality when selecting imaging modalities for each individual patient and be knowledgeable when interpreting the examination results.

Severe organ ischemic damage can be lethal to patients with TAK. Some patients may develop life-threatening aneurysms or arterial dissections due to uncontrolled active disease or complications. Patients with severe heart valve diseases can develop heart failure. Acute coronary events and stroke are also the major causes of death. Patients who developed these vital organ damage not only need medical treatment, but may also need emergent or elective surgical or endovascular interventions. Peri-operative medical treatment, including adjusting the dosage of glucocorticoid and immunosuppressive agents to control active inflammation, and anti-platelet treatment and/or anticoagulant management are critical to reduce the risk of peri-operative complications and mortality. So multidisciplinary management of patients with TAK has been proven to be an effective approach to optimize treatment outcomes. Lack of multidisciplinary collaboration may result in high rates of surgical complications and mortality as well as high rates of restenosis. Therefore, multidisciplinary collaboration should be strengthened.

European League Against Rheumatism (EULAR) endorsed the recommendations for the management of large vessel vasculitis in 2018 and American College of Rheumatology (ACR) developed the guideline for the management of large vessel vasculitis in 2021. These guidelines pointed out that treatment of TAK could be divided into remission induction and remission maintenance stages. The basic treatment for remission induction is high dose glucocorticoid combined with traditional immunosuppressive agents, while for remission maintenance, glucocorticoid should be tapered to the minimum maintenance dose or stopped, but immunosuppressive agents should be combined to maintain the disease in a persistent stable state. However, it is not seldom that patients were treated with glucocorticoid only in China. Many patients developed steroid induced diabetes mellitus or atherosclerosis due to long-term use of glucocorticoid. Biological agents have been used more and more frequently in treating active TAK, but due to the limited evidence, they are recommended to treat relapsed or refractory disease by the guidelines. However, some rheumatologists prescribe biological agents as the initial treatment of TAK. This leads to increased medical costs. Therefore, the level of standardization of treatment should be improved.

Unlike other common rheumatic diseases such as systemic lupus erythematosus and rheumatoid arthritis, there is very few high-quality clinical studies on TAK worldwide. Most of the existing studies are retrospective observational and case series studies. Most of the studies on TAK treatment are case reports or small sample, retrospective, single-center studies. So far, there are only two randomized double-blind controlled studies on TAK drug therapy. ^[6,7]^ The overall quality of TAK research is low. Therefore, large-scale high-quality clinical studies is mandatory to provide strong evidence to support treatment strategy.

In summary, TAK is a rare and poorly studied disease worldwide. The heterogeneity and the complexity of the disease make the management of TAK very challenging. Low early diagnosis rate, low level of standardized treatment, high incidence of major complications and poor overall prognosis are the 4 major problems in the management of TAK. The National Clinical Research Center for Dermatologic and Immunologic Diseases (Peking Union Medical College Hospital) developed and released the first Chinese guideline for the diagnosis and treatment of Takayasu’s arteritis (2023)^[8]^ in an evidence based approach. This guideline also takes the published international guidelines on TAK as important references. This guideline covers 11 clinical scenarios that are important to the diagnosis and treatment as well as long-term management of TAK. It also provides measures to deal with the above 4 problems and challenges in the management of TAK based on the best evidence from the literature. We believe that the release of this guideline will greatly help in improving the current status of the diagnosis and treatment of TAK not only in China, but around the world. It can also help in improving the standardization of the treatment of TAK and hopefully improve the overall prognosis of patients with TAK in China. We also hope that this guideline will provide instruction for physicians who take care of patients with TAK in countries and areas where TAK is prevalent.
